# Relationship between Capillaroscopic Architectural Patterns and Different Variant Subgroups in Fabry Disease: Analysis of Cases from a Multidisciplinary Center

**DOI:** 10.3390/genes15081101

**Published:** 2024-08-21

**Authors:** Denise Cristiana Faro, Francesco Lorenzo Di Pino, Margherita Stefania Rodolico, Luca Costanzo, Valentina Losi, Luigi Di Pino, Ines Paola Monte

**Affiliations:** 1 Department of Surgery and Medical-Surgical Specialties, University of Catania, 95125 Catania, Italy; denisecristiana.faro@unict.it (D.C.F.); francesco.dipino@icloud.com (F.L.D.P.); vale.losi@gmail.com (V.L.); dipino@unict.it (L.D.P.); 2 Institute for Biomedical Research and Innovation, National Research Council (IRIB-CNR), Section of Catania, 95126 Catania, Italy; margheritastefania.rodolico@cnr.it; 3 Unit of Angiology, Policlinico “G. Rodolico-San Marco” University Hospital, 95123 Catania, Italy; lucacost84@gmail.com; 4 Unit of Cardiology, “G. Rodolico-S.Marco” University Hospital, 95123 Catania, Italy

**Keywords:** Anderson–Fabry disease, endothelial dysfunction, rare diseases, lysosomal storage, capillaroscopy, intima-media thickness, inflammation pathways, X-linked

## Abstract

Anderson–Fabry disease (AFD) is a genetic lysosomal storage disorder caused by mutations in the α-galactosidase A gene, leading to impaired lysosomal function and resulting in both macrovascular and microvascular alterations. AFD patients often exhibit increased intima-media thickness (IMT) and reduced flow-mediated dilation (FMD), indicating non-atherosclerotic arterial thickening and the potential for cardiovascular events. Nailfold capillaroscopy, a non-invasive diagnostic tool, has shown potential in diagnosing and monitoring microcirculatory disorders in AFD, despite limited research. This study evaluates nailfold capillaroscopy findings in AFD patients, exploring correlations with GLA gene variant subgroups (associated with classical or late-onset phenotypes and variants of uncertain significance (VUSs)), and assessing morpho-functional differences between sexes. It aims to determine whether capillaroscopy can assist in the early identification of individuals with multiorgan vascular involvement. A retrospective observational study was conducted with 25 AFD patients from AOUP “G. Rodolico-San Marco” in Catania (2020–2023). Patients underwent genetic testing, enzyme activity evaluation, and nailfold capillaroscopy using Horus basic HS 200 videodermatoscopy. Parameters like angiotectonic disorder, vascular areas, capillary density, and intimal thickening were assessed. The study identified significant differences in capillaroscopy findings among patients with different GLA gene variant subgroups. Classic AFD variant patients showed reduced capillary length and signs of erythrocyte aggregation and dilated subpapillary plexus. No correlation was found between enzymatic activity and capillaroscopy parameters. However, Lyso-Gb3 levels were positively correlated with average capillary length (ῤ = 0.453; *p* = 0.059). Sex-specific differences in capillaroscopy findings were observed in neoangiogenesis and average capillary length, with distinct implications for men and women. This study highlights the potential of nailfold capillaroscopy in the diagnostic process and clinical management of AFD, particularly in relation to specific GLA gene mutations, as a valuable tool for the early diagnosis and monitoring of AFD.

## 1. Introduction

### 1.1. Fabry Disease Overview

Anderson–Fabry disease (#301500) is an X-linked lysosomal storage disorder caused by pathogenic variants in the α-galactosidase A gene (*GLA*, Gene Entrez: 2717; NCBI reference sequence: NM_000169.3; *300644; Locus Reference Genomic record LRG_672), responsible for encoding the enzyme α-galactosidase A (α-Gal A, EC 3.2.1.22; Uniprot P06280), resulting in reduced or absent enzyme activity and impaired lysosomal function [1,2]. As a monogenic X-linked recessive hereditary disease, it has an estimated birth prevalence of 1:40,000–117,000 [3,4]. Hemizygous males typically exhibit full disease features due to nearly absent or markedly reduced α-galactosidase A activity, with symptoms spanning neurological, dermatological, renal, cardiovascular, cochleo-vestibular, and cerebrovascular areas [5,6]. Heterozygous females, once considered mere carriers, can have symptoms ranging from very mild to severe due to lyonization, which causes cellular mosaicism [7,8,9]. Lyonization, or X-chromosome inactivation, occurs randomly in females, leading to differences in gene expression between cells expressing the wild-type or mutated allele.

The disease usually begins in childhood or even in fetal development [10], and unlike many other lysosomal storage diseases, patients often remain asymptomatic in early years [11]. The disease’s progression involves organ damage linked to vascular endothelial and smooth muscle cells, particularly in microcirculation, cardiomyocytes, renal epithelial cells, and neuronal cell types [2,12]. Secondary pathological processes, including inflammation, ischemia, hypertrophy, and fibrosis, can also contribute to organ damage and fragility.

Fabry disease presents two distinct phenotypes: classic and non-classic, or late-onset. In classic Fabry disease, often seen in hemizygous males with no residual α-galactosidase A activity, common signs include burning pain, hypo- or hyperhidrosis, transient ischemic attacks, stroke, cutaneous angiokeratoma, proteinuria, cardiomyopathy, cardiac arrhythmias, and gastrointestinal disturbances. Patients with non-classical disease possess residual enzyme activity (2–20% of normal) and exhibit a milder form with symptoms typically appearing later in life, often limited to a single organ system [13].

Genetic variants in Fabry disease are classified into the following categories: pathogenic, likely pathogenic, VUSs (variants of uncertain significance), likely benign, and benign according to the American College of Medical Genetics and Genomics guidelines [4,14]. The genotype–phenotype correlation is complicated by the disease’s rarity, allelic heterogeneity, and variation in clinical expressivity, compounded by a lack of comprehensive clinical data [15].

Fabry disease’s natural course can be significantly altered by enzyme replacement therapy (ERT). Introduced in Europe in 2001 and in the United States in 2003, recombinant human α-galactosidase A is available in two forms: agalsidase alpha and agalsidase beta. Since 2016, chaperone therapy with migalastat has been an option, stabilizing residual endogenous enzyme present but not bioavailable due to misfolding. This orally administered drug is indicated for patients with susceptible missense mutations [16]. In addition, Pegunigalsidase alfa, a PEGylated ERT, formulation has shown efficacy in reducing plasma lyso-Gb3, placing it as a suitable, less immunogenic alternative to current ERT options [17,18,19].

### 1.2. Fabry Disease as a Vascular Systemic Pathology

The disease primarily manifests as a systemic vascular disease causing cardiovascular complications. Several mechanisms, including occlusion and luminal obstruction due to GL-3 accumulation in vascular endothelial cells, the disruption of vasodilators and vasoconstrictors balance, and thromboembolic complications, contribute to ischemic tissue damage [20].

Fabry vasculopathy involves abnormalities in blood components, blood flow, and vascular walls (Virchow’s triad), leading to vascular dysfunction. Lysosomal Gb3 accumulation in arterial wall cells leads to changes in endothelial and smooth muscle cells, fibrotic thickening, and endothelial dysfunction [21,22]. This process results in vascular lesions involving endothelial and cerebral perfusion alterations and a prothrombotic phenotype [23,24]. Cardiovascular risk factors may exacerbate arterial performance decline [25]. Gb3 accumulation activates the local renin–angiotensin system, upregulating adhesion molecules and prothrombotic factors while decreasing nitric oxide synthesis [22]. It also induces reactive oxygen species production and endothelial nitric oxide (NO) synthase dysregulation, leading to muscular hypercontractility and vasospasm [26,27,28].

Gb3 reduces calcium-activated potassium channel 3.1 in endothelial cells, inhibiting endothelium-derived relaxing factor production [29,30]. Lyso-Gb3, which promotes smooth muscle cell proliferation, is elevated in young male AFD patients, suggesting early arterial involvement [22,31]. Increased intima-media thickness (IMT) in AFD, a specific vascular change, differs from traditional IMT alterations in atherosclerotic disease [22].

Hyperdynamic circulation and less elastic vascular walls may over-regulate the local renin–angiotensin system. Angiotensin II, through AT1 receptor, triggers an inflammatory cascade affecting adhesion molecule and chemokine expression [32,33]. Elevated levels of soluble sICAM-1, sVCAM-1, P-selectin, PAI, and decreased thrombomodulin with increased CD11b monocyte expression indicate a prothrombotic state [24].

ROS production in Fabry disease, possibly related to glycolipid accumulation, affects endothelial caveolar function and mechanotransduction [26]. Lyso-Gb3, elevated in Fabry patients, inhibits α-galactosidase A and promotes smooth muscle cell proliferation, correlating with increased intima-media thickness and cardiac mass in heterozygous females [31,34]. Cerebral circulation dysfunction in Fabry patients, particularly significant hyperperfusion in posterior regions, suggests heterogeneity in response to glycolipid accumulation [23].

Peripheral neuropathy reflects the deterioration of neuronal cells in the autonomic and somatosensory peripheral nervous system due to Lyso-Gb3 deposits. Hypohidrosis, a sign of selective damage to peripheral nerves, and gastrointestinal manifestations are linked to Lyso-Gb3 accumulation in various cells and structures [35,36]. Renal failure in Fabry disease, beyond accumulation material, includes glomerulosclerosis and proteinuria. Fabry proteinuria not responsive to enzyme replacement infusions may be associated with cathepsin-L overexpression and altered dynamin processing, potentially reversible with appropriate treatment [37].

Fabry disease is characterized by macrovascular and microvascular alterations [38]. Studies reveal that AFD patients exhibit increased IMT without significant plaques in the carotid, brachial, and abdominal aorta, and reduced flow-mediated dilation (FMD) in the brachial artery compared to healthy individuals [39,40,41]. These changes are markers of generalized atherosclerosis and are associated with traditional cardiovascular risk factors such as aging, high LDL cholesterol, hypertension, smoking, type 2 diabetes, and microalbuminuria [42,43].

In terms of microvascular alterations, AFD leads to endothelial Gb3 deposits in small, medium, and large vessel walls, leading to classic cardiac, renal, and cerebrovascular symptoms. Specific nailfold capillary changes such as atypical bushy or clustered capillaries have been observed in AFD patients [44,45,46,47]. Glycosphingolipid accumulation induces small fiber neuropathy (autonomic and sensory), endothelial dysfunction, and smooth muscle cell proliferation, potentially affecting vascular tone and triggering Raynaud’s phenomenon. This phenomenon may partly cause extremity pain in nearly half of Fabry patients [44,45]. These alterations reflect the underlying endothelial dysfunction and altered terminal organ perfusion due to arteriovenous shunting and capillary inadequacy. Costanzo et al.‘s observational study showed that AFD mutation carriers, even asymptomatic, had twice the rate of irregular capillary architecture and significantly different atypical capillaries than controls [46]. Nailfold capillaroscopy (NC) has been recognized as a valuable diagnostic tool for assessing these microvascular changes and guiding early treatment strategies.

## 2. Materials and Methods

### 2.1. Study Purpose

Nailfold capillaroscopy is a simple, rapid, painless, and non-invasive diagnostic tool useful in diagnosing and monitoring microcirculatory disorders. While the literature on its use in AFD diagnosis and monitoring is still limited by sample sizes and study numbers, it suggests a significant role for this technique. AFD, primarily considered a progressive microcirculatory pathology, is also characterized by macrovascular involvement. Numerous studies have investigated arterial involvement using carotid ultrasound evaluation. This study aims to evaluate nailfold capillaroscopy findings in a cohort of Fabry disease patients, analyze correlations with GLA gene mutation types in the sample, and assess morpho-functional alteration differences between sexes. Furthermore, it explores whether capillaroscopy findings can aid in early identification of individuals with multiorgan micro- and macrovascular involvement. To our knowledge, this is the first study examining such relationships in a cohort categorized by GLA gene mutation types.

### 2.2. Study Design and Procedures

This retrospective observational study involved 25 patients, selected from 72 attending the Clinical Echocardiography, Cardioncology, and Rare Cardiomyopathies Lab, and Angiology Lab—Cardio-Thoracic-Vascular and Transplants Department, CAST of AOUP “G. Rodolico-San Marco” in Catania, between 2020 and 2023. Inclusion criteria were:

- Age over 18.

- AFD diagnosis (symptomatic/asymptomatic), starting from clinical suspicion or extended family screening, genetically confirmed by identifying GLA gene pathogenic variants in peripheral blood samples of consenting participants.

- Classic or non-classic phenotype, associated with pathogenic (classic, non-classic) variants and VUS; polymorphisms considered benign according to recent international consensus were excluded.

Patients’ demographic, clinical, and laboratory data, therapeutic regimens, and capillaroscopy findings were collected from medical records. AFD diagnosis was made by analyzing GLA gene pathogenic variants using real-time PCR in “high-resolution melting”; α-Gal A enzyme activity and lyso-Gb3 levels were evaluated at IRIB-CNR laboratories. GLA gene variants were classified as per American College of Medical Genetics and Genomics guidelines into pathogenic, likely pathogenic, VUS, likely benign, and benign categories.

All patients underwent supra-aortic trunk echocolor Doppler examination, and common carotid artery IMT data were recorded. IMT was measured using a Mylab Seven Esaote echotomograph with an L3-11 linear probe by two trained sonographers. B-mode ultrasound detection was performed on the far wall from the skin surface in both carotid arteries, and the widest IMT measurement was used for analysis. Longitudinal scans of the common carotid artery (CCA) were performed 2 cm proximal to the bifurcation.

#### Nailfold Capillaroscopy: Methodology

In our study, we adhered to established protocols to ensure the reliability and accuracy of our capillaroscopic assessments, consistent with best practices as described in the literature [48,49,50,51,52].

Before the capillaroscopic examination, all patients were acclimatized in a room maintained at a constant temperature of 20–25 °C for at least 15–20 min to avoid any temperature-induced changes in microcirculation. This step is critical as exposure to cold can cause significant vasoconstriction, which may alter capillary appearance.

We also instructed patients to refrain from caffeine intake and smoking for 4–6 h prior to the examination to minimize any vasoconstrictive effects these could induce. Furthermore, patients were advised not to remove their fingernail cuticles for at least one month before the examination to prevent any microtraumas that could impact the capillaroscopic findings.

The eligibility criteria for capillaroscopy included the exclusion of patients with recent finger injuries or any condition that could affect the nailfold area. Additionally, we ensured that the patients’ hands were clean, and a drop of vegetable oil was applied to improve the visualization of capillaries during the examination.

These rigorous procedures were implemented to minimize potential confounders and to ensure that the capillaroscopic images obtained were both reliable and reflective of the true microcirculatory status of the patients.

Nailfold capillaroscopy was performed on the last three fingers of each hand using a Horus basic HS 200 high-definition videodermatoscopy system. Capillary morphology was independently assessed by two experienced operators according to previously reported AFD models. Disagreements were resolved by consensus.

Parameters evaluated included:

- Angiotectonic disorder;

- Vascular and pseudovascular areas;

- Capillary density;

- Heterogeneity;

- Giant capillaries;

- Microhemorrhages;

- Ectasias;

- Average capillary length;

- Loop tortuosity;

- Thrombosed loops;

- Acrosyndrome;

- Neoangiogenesis;

- Flow;

- Sub-papillary plexus.

### 2.3. Ethical Aspects

The study was conducted in accordance with the Declaration of Helsinki and Good Clinical Practice guidelines, with the approval of the local ethics committee.

Data were de-identified before statistical analysis to respect patient privacy and were reported anonymously and aggregated. Subjects at our multidisciplinary center were subjected to informed consent prior to the execution of genetic tests and consented to the handling of their data in an anonymous and aggregated form for research purposes. Patients also provided written informed consent before genetic testing.

### 2.4. Statistical Analysis

Statistical analysis was conducted using Medcalc and IBM-SPSS v.26.0 packages. Categorical variables were described in terms of absolute frequency and prevalence, while continuous variables were further divided into two groups after the Kolmogorov–Smirnov test to assess distribution. They were expressed as mean ± standard deviation for normally distributed variables and median with interquartile range for non-Gaussian variables. Differences in demographic, clinical, and laboratory data between the two populations were evaluated using Fisher’s exact Chi-squared test for categorical variables, Mann–Whitney U test for non-Gaussian continuous variables, and Student’s *t*-test (and ANOVA for more than two groups) for normally distributed continuous variables. For correlation studies of the variables of interest, non-parametric tests like Spearman’s correlation coefficient and Kendall’s Tau were necessary.

Where appropriate, these correlations were evaluated using partial correlation tests. Univariate and binary logistic regression analysis was also used, as appropriate, based on variables to be tested. Significant relationships were further analyzed using multivariate logistic and linear regression models. The goodness of fit for logistic models was assessed using the Hosmer and Lemeshow test. The null hypothesis was rejected in all two-tailed tests for *p*-values < 0.05.

## 3. Results

This study evaluated capillaroscopy findings in 25 AFD patients.

**Figure 1:**
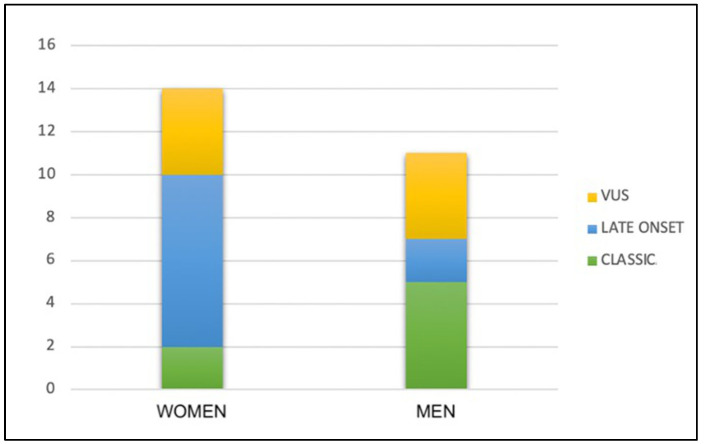
Distribution of GLA variant subgroups in the study population.

**Figure 2:**
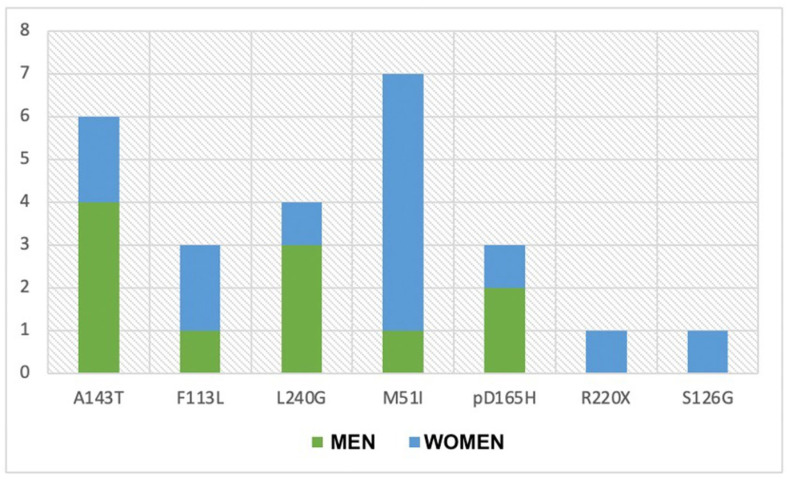
Distribution of specific genetic variants in male and female patients.

Patients were divided into three subgroups based on the mutational variant of the GLA gene, which include classical- and late-onset-phenotype-associated variants, categorized into two subgroups, and the VUS subgroup (Figure 1 and Figure 2).

Demographic, laboratory characteristics, and morpho-functional alterations observed in capillaroscopy are shown in Table 1.

Regarding demographics, age in the three groups (classic, late-onset, and VUS) was homogeneous and comparable; male prevalence showed no significant differences within subgroups. Statistically significant differences were observed in average Lyso-Gb3 levels; patients with the classic variant showed a statistically higher accumulation of Lyso-Gb3 compared to those with late-onset or VUSs, corresponding to reduced enzymatic activity in the classic variant group.

**Figure 3:**
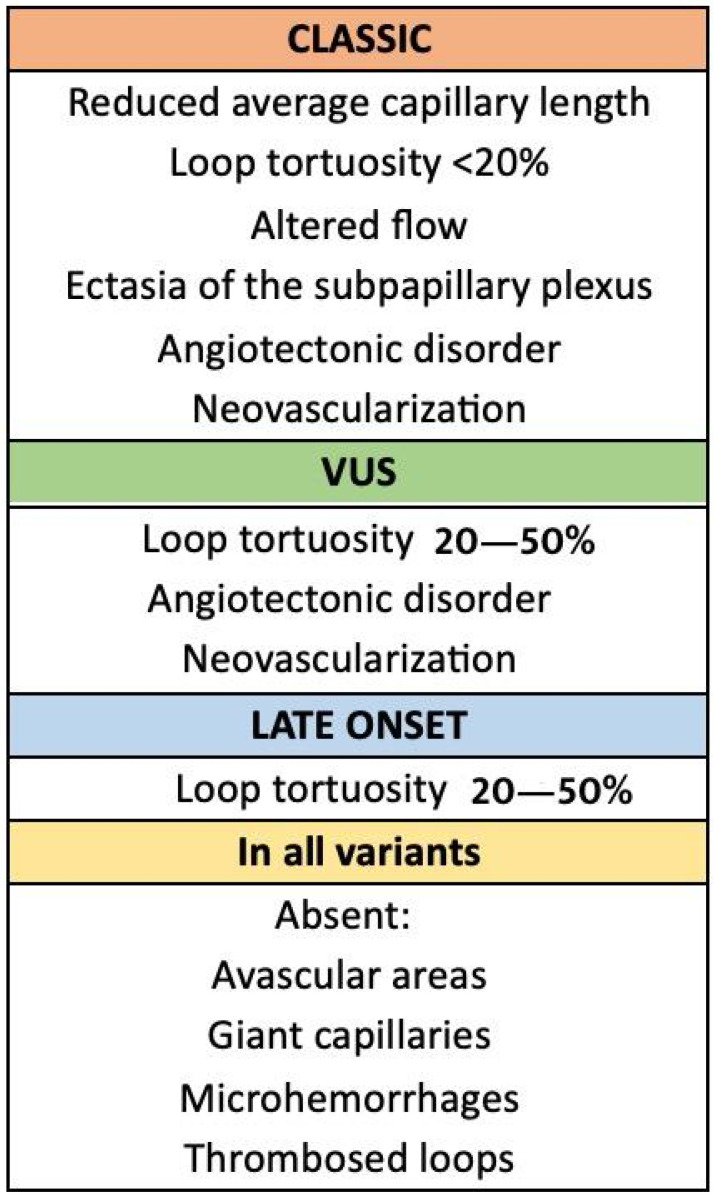
Summary of capillaroscopic findings according to GLA.

Patients with the classic AFD variant had significantly reduced average capillary length, less than 20% tortuosity, erythrocyte aggregation presence, and dilated subpapillary plexus. Patients with late-onset variants had an invisible subpapillary plexus as a statistically significant variable in the hypothesis test and 20–50% tortuosity, also significant in VUS patients. These patients showed a significant absence of angiotectonic disorder compared to other subgroups. No significant differences were observed in other considered parameters like edema, giant capillaries, vascular and pseudovascular areas, acrosyndrome, microhemorrhages, and capillary heterogeneity. Neoangiogenesis was significantly less present in VUS patients (*p* = 0.029) (Figure 3).

**Figure 4:**
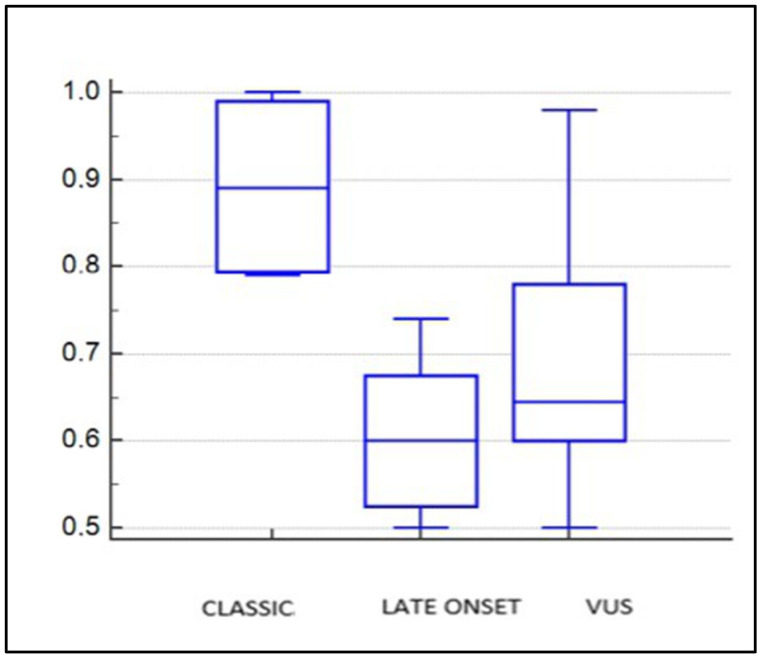
One-way ANOVA: intima-media thickness in right common carotid in sub-groups.

Regarding intimal thickening, assessed by echocolor Doppler TSA, patients with the classic AFD variant had significantly higher right carotid IMT compared to other mutation subgroups, with an IMT value of 0.89 (Table 2, Figure 4). No significant differences were observed in the left common carotid intimal thickness.

Potential correlations between enzymatic activity and capillaroscopy and echocolor Doppler TSA findings that were significant in the hypothesis tests were also analyzed. No correlation was observed between alpha-galactosidase levels and capillaroscopy parameters, while Lyso-GB3 levels positively correlated with average capillary length (ῤ = 0.453; *p* = 0.059).

**Figure 5:**
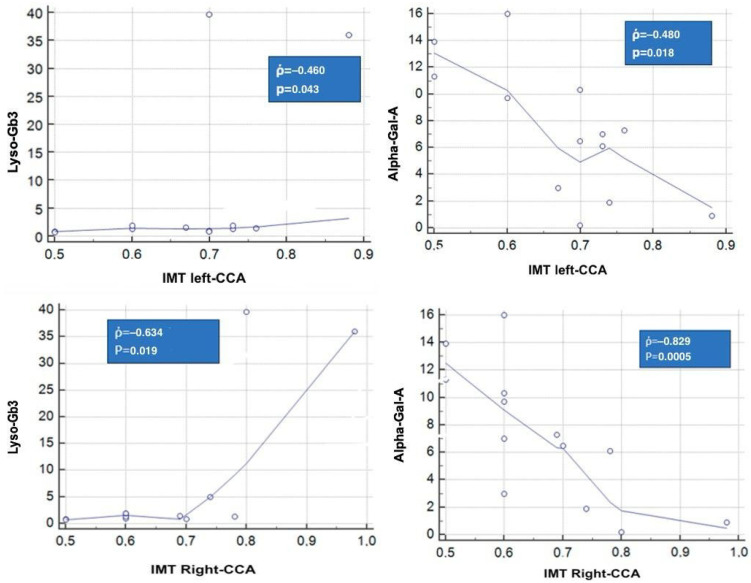
Correlation between alfa-gal A, Lyso-Gb3, and IMT levels. Abbreviations: see in the text.

Regarding intimal thickening, alpha-galactosidase and Lyso-GB3 levels correlated with right and left carotid IMT (Figure 5).

**Figure 6:**
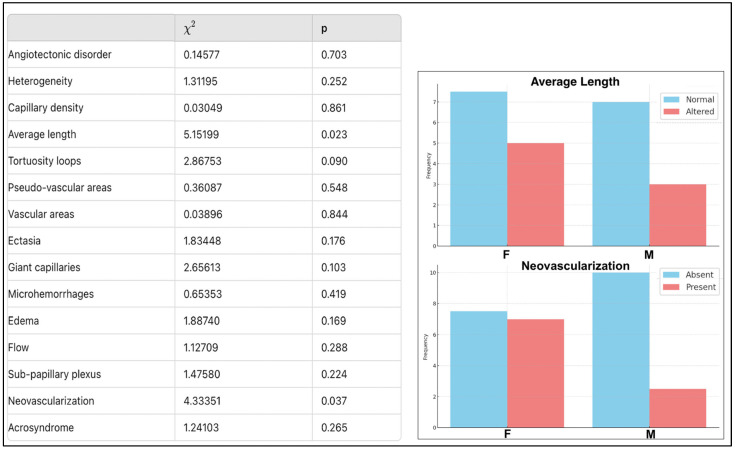
Comparison of nailfold capillaroscopy findings in men and women from our sample. The figure shows greater alteration in capillary length in males (M), while there is more neovascularization in females (F), in the absence of other statistically significant differences.

Using logistic and linear regression analyses for ordinal variables, various models were constructed with the significant capillaroscopy findings as the dependent variables and age, sex, alpha-galactosidase, Lyso-Gb3 levels, and the GLA gene variant subgroup as covariates in a stepwise manner. The classic AFD variant was associated with reduced average length (OR = 65, 95% CI 3.37–1251.28; *p* = 0.005), the absence of over 20% tortuosity (OR 0.090, 95% CI 0.009–0.879; *p* = 0.0384), over 30% ectasia presence (OR 14, *p* = 0.0453), and erythrocyte aggregation (OR 30; 95% CI 2.1–411, *p* = 0.010), regardless of the aforementioned confounding factors. The data regarding invisible subpapillary plexus significantly present in patients with VUS and late onset were not confirmed in logistic regression analysis; the only variable considered in the model was the Lyso-Gb3 value (OR = 4, *p* = 0.032). Finally, potential differences in capillaroscopy parameters between sexes were evaluated (Figure 6).

**Figure 7:**
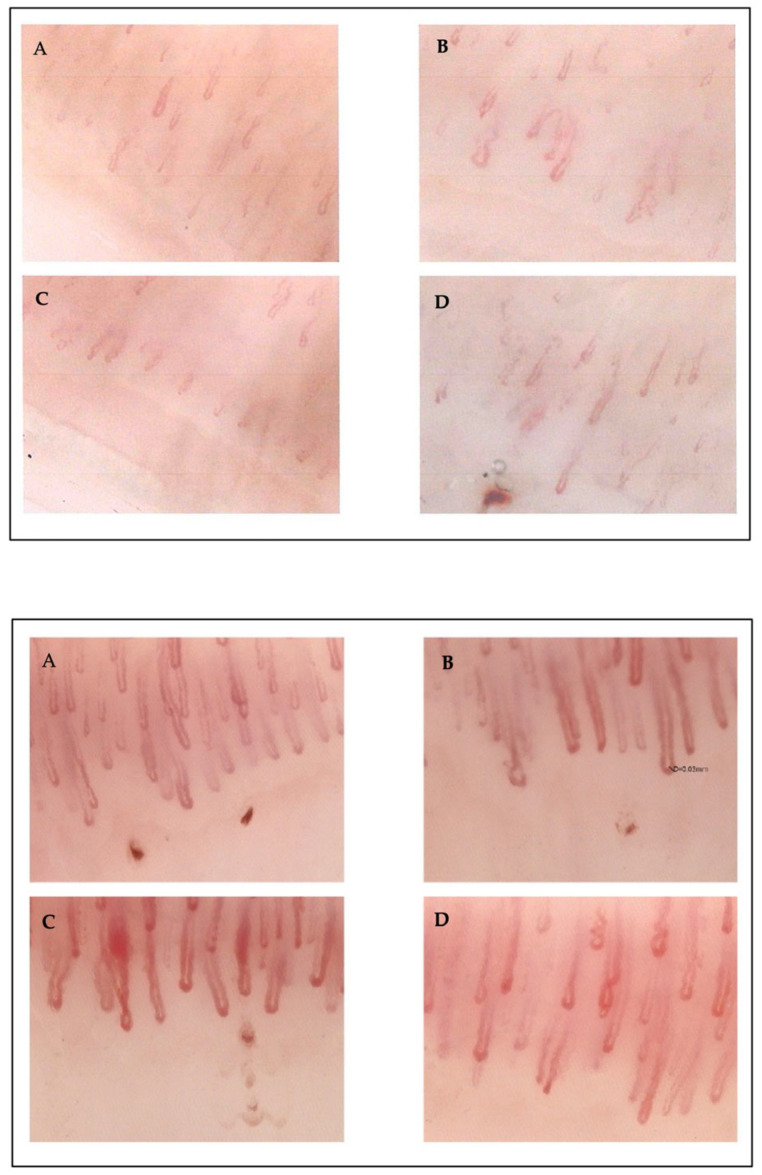
*Upper*: Capillaroscopic picture in a 71-year-old female patient with a classic Fabry variant, showing moderate angiotectonic disorder and heterogeneity, reduced capillary density (**A**,**B**), increased average length, limited pseudovascular areas, 10–30% ectasias, loop tortuosity of 20–50%, rare microhemorrhages, and moderate neoangiogenesis (**C**,**D**). *Bottom:* Male, 45 years old, VUS, predominantly ischemic Raynaud’s phenomenon. The picture is characterized by diffuse anomalies in capillary architecture and morphology (**A**,**B**), widespread capillary ectasias, isolated mega capillaries, rare microhemorrhages, and neoangiogenesis (**C**,**D**).

Significant differences were observed between the two sex-differentiated subgroups in neoangiogenesis, more prevalent in women, and altered average capillary length, more altered in men. Generally, we can state that men and women exhibit different but equally severe capillaroscopic alterations (Figure 7).

Exploratory analysis according to therapy status.

We conducted an exploratory analysis to evaluate the impact of AFD-specific treatment on various microcirculatory parameters and clinical complications in our patient cohort, acknowledging the limited sample size for treated patients, which may likely affect the statistical significance of the findings. Given the additional difficulty of further dividing the data into subgroups based on the type of therapy, we compared a combined group of “treated” patients (on ERT or migalastat) with those not receiving therapy across multiple variables. Nevertheless, in an explorative way, we also compared patients on ERT with those on Migalastat and untreated ones.

For microcirculatory characteristics, we compared all the available NFC parameters in the groups. The results revealed a statistically significant difference between the two groups only in “sub-papillary plexus” (*p* = 0.036). Other variables, including capillary flow (*p* = 0.133), inhomogeneity (*p* = 0.399), capillary density (*p* = 0.357), capillary tortuosity (*p* = 0.514), pseudovascular areas (*p* = 0.789), ectasias (*p* = 0.475), giant capillaries (*p* = 0.276), microhemorrhages (*p* = 0.568), edema (*p* = 0.227), thrombotic loops (*p* = 0.748), neoangiogenesis (*p* = 0.906), avascular areas (*p* = 0.390), and acrosyndrome (*p* = 0.562), did not show statistically significant differences.

Further analysis was performed to differentiate between patients on ERT, those on migalastat, and those not receiving any therapy. The ANOVA results indicated that most variables did not differ significantly across the three groups. However, the “sub-papillary plexus“ variable approached statistical significance (*p* = 0.079), suggesting a potential trend in the difference between the groups, though this did not reach the level of significance.

Regarding clinical complications, we examined variables such as hearing loss, cardiac ischemia, stroke or TIA, cardiac hypertrophy, and retinal vasculopathy. No statistically significant differences were observed between the groups for any of these complications, with *p*-values ranging from 0.114 to 0.269.

**Figure 8:**
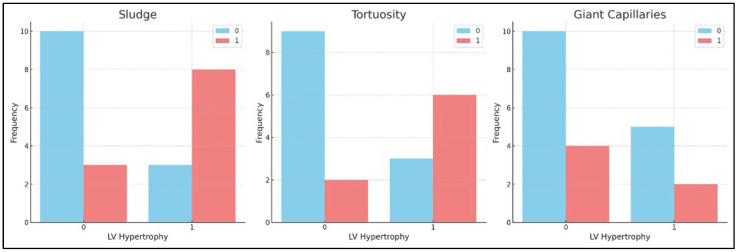
Relation between capillaroscopic alterations and left ventricular hypertrophy in the study population. Legend: “0 (blue bar)” means that the alteration is “absent” and “1 (red bar)” means that the alteration is present.

In addressing the final question of our study, we sought to determine whether capillaroscopic findings could be associated with other microvascular and macrovascular manifestations. Erythrocyte aggregation phenomena (sludge), loops with tortuosity greater than 20%, and the presence of giant capillaries were more significantly observed in patients with hearing loss and LV hypertrophy. (Figure 8).

## 4. Discussion

This study aimed to investigate nailfold capillaroscopy findings and eco-color Doppler examinations of the supra-aortic trunks in a cohort of patients with Anderson–Fabry disease, analyzing potential correlations with GLA gene mutation types, as a targeted diagnostic tool for improving early detection of the disease and optimizing continuous monitoring of patients.

The results revealed significant capillaroscopic differences among patients with different GLA gene variant subgroups. Patients with the classic variant of AFD showed more significant capillaroscopic alterations than other variants: notably, reduced average capillary length, tortuosity of the loops under 20%, altered flow, erythrocyte aggregation, dilated subpapillary plexus, significantly increased ectasia, angiotectonic disorder, and neovascularization. Patients with VUSs predominantly exhibited loop tortuosity of 20–50%, angiotectonic disorder, and neovascularization. Patients with late-onset variants primarily exhibited loop tortuosity <20% and a non-visible subpapillary plexus. Across all genetic variant subgroups, there were no detected cases of avascular areas, giant capillaries, microhemorrhages, or thrombosed loops.

Endothelial dysfunction in AFD has been widely described in the literature with in vitro and in vivo studies. Potential mechanisms include Gb3 accumulation in the endothelium, smooth muscle cell proliferation, increased IMT, heightened endothelial activation markers, a phenotypic shift towards a prothrombotic state, and reduced NO bioavailability.

In AFD, a morphological and functional microangiopathy of the nailfold capillaries is evident. The first identification of dystrophic capillaries in AFD occurred in 1993, followed by additional case studies and series that, although limited in number, described capillaroscopic abnormalities in AFD patients and agreed that capillaroscopic results based on microcirculation studies can provide valuable information in pathophysiology, differential diagnosis, and therapy monitoring.

Our findings align with previous studies in the field. The consistent observation of increased IMT and reduced FMD across multiple studies highlights the systemic vascular involvement in Fabry disease. For instance, Rombach et al. observed an increase in IMT and pulse wave velocity in the common carotid arteries and femoral arteries of AFD patients [53]. Their results showed a 9% increase in IMT for males and 8% for females, along with significant reductions in FMD, especially in males (−30%). Similarly, Wasik et al. noted thick capillaries and other pathological patterns in AFD patients, indicating microangiopathy through capillaroscopy [44]. Kalliokoski et al. described increases in IMT in multiple vessels, including the CCA and brachial artery, and the most considerable differences in abdominal aorta IMT, along with significant reductions in FMD. Their study revealed a 27% increase in aortic IMT and a 33% reduction in FMD, indicating extensive vascular involvement, aligning with McGill et al.’s findings that atherosclerotic lesions often initiate in this area [39,54].

The consistent observation of increased IMT across different studies highlights the pervasive impact of Fabry disease on the vascular system, while the thickening and structural changes in capillaries reflect the underlying endothelial dysfunction and deposition of glycolipids, which are central to Fabry disease pathophysiology [46]. Our study’s findings of capillaroscopic alterations, especially in patients with the classic variant of AFD and increased IMT, align with these results, underscoring the systemic vascular involvement in AFD and further supporting the utility of capillaroscopy in identifying early vascular changes in Fabry patients.

Our study observed a significant increase in right common carotid IMT, although remaining within physiological dimensions. IMT is a marker of organ damage and is correlated with multi-organ damage. The literature indicates a significant increase in common carotid artery IMT in Fabry patients occurring in the absence of focal atherosclerotic plaques.

Boutouyrie et al. observed accelerated arterial wall hypertrophy in medium-sized vessels in AFD patients, particularly a 2.3-fold increase in IMT in the radial artery where IMT widened more rapidly than in healthy controls [41]. A previous study by Costanzo et al. on Fabry disease mutation carriers found a 23% increase in carotid IMT without plaques and a 32% reduced brachial artery FMD, along with significant microangiopathy in nailfold capillaries, indicating early macrovascular involvement [46]. Barbey et al. found a 13% increase in IMT in male AFD patients and an 18% increase in females without the presence of plaques, revealing that only age correlated with common carotid artery IMT [34,43]. This aligns with our findings of increased IMT in the absence of atherosclerotic plaques, suggesting that the arterial thickening in Fabry disease is primarily due to glycolipid deposition rather than traditional atherosclerotic processes and emphasizing the importance of monitoring arterial thickening as a marker of vascular involvement in Fabry disease [40,41].

Increased carotid IMT has been shown to predict coronary atherosclerosis, coronary disease, and stroke development, even after adjusting for other cardiovascular risk factors [55,56,57], serving as a surrogate marker for generalized atherosclerosis [42]. Similar alterations in radial artery IMT, a medium-sized muscular artery, were also noted, a phenomenon unique to AFD among lysosomal storage disorders [40,58]. Most AFD patients in Barbey et al.‘s study had traditional cardiovascular risk factors contributing to notable carotid wall thickening [43]. Interestingly, despite increased IMT, no carotid bifurcation plaques were observed in these patients. Preserved or improved vasomotricity and vascular elasticity in AFD might represent adaptive changes providing apparent “cardioprotection” against the enzymatic defect [40].

Limited data exist on early cardiovascular damage in Fabry disease without cardiac hypertrophy. Studies by Costanzo and Monte et al. aimed to evaluate cardiac macrovascular and microvascular functions in Fabry mutation carriers without hypertrophy. They found preclinical cardiac function impairments using TDI and longitudinal deformation, early macrovascular involvement through IMT and FMD assessments, and microvascular alterations via capillaroscopy [46]. These results underscore early myocardial function anomalies and vascular system involvement in mutation carriers. AFD is associated with marked non-atherosclerotic arterial thickening. Evidence suggests increased IMT as a future cardiovascular event indicator, and preventive measures can reduce complication incidence. Whether carotid artery IMT stabilization or regression is a valid treatment efficacy indicator in AFD patients remains an area for further clinical research.

Our study adds to this body of knowledge by demonstrating significant capillaroscopic differences among patients with different GLA gene variant subgroups. The pronounced capillaroscopic changes in patients with the classic variant of AFD suggest more severe microvascular involvement, contributing to the greater clinical severity observed in these patients. The findings from Wasik et al. and Costanzo et al. further support the utility of capillaroscopy in identifying early vascular changes in Fabry patients [44,46]. The presence of thick capillaries and other pathological patterns observed in these studies and corroborated by our findings reflect the underlying endothelial dysfunction and deposition of glycolipids that are central to Fabry disease pathophysiology.

Wasik et al.‘s case–control study explored NFC as an additional diagnostic tool, revealing a significant presence of atypical capillaries, primarily bushy or clustered, compared to healthy controls. Other connective tissue disease characteristics like giant capillaries, avascular fields, and irregular architecture were not prevalent in AFD patients. Altered endothelial function due to intracellular glycosphingolipid accumulation and Stemper and Hilz’s hypothesis of altered terminal organ perfusion due to arteriovenous shunting and capillary inadequacy might explain NFC alterations [44,59]. Interestingly, nailfold capillary morphological changes were similar in hemi- and heterozygous Fabry patients and not age-dependent. Despite renal failure being a common complication in advanced AFD stages, no severe microangiopathy was noted in patients with significant nephropathy. The high incidence of Raynaud’s phenomenon, previously unreported, should be considered a cause of secondary Raynaud’s phenomenon in AFD. Glycosphingolipid accumulation induces small fiber neuropathy (autonomic and sensory), endothelial dysfunction, and smooth muscle cell proliferation, potentially affecting vascular tone and triggering Raynaud’s phenomenon. This phenomenon may partly cause extremity pain in nearly half of Fabry patients [44,45].

Deshayes et al. highlighted a higher prevalence of Raynaud’s phenomenon in AFD patients, particularly in males, with a 38% increase compared to controls, suggesting that microcirculatory dysfunction is a significant aspect of Fabry disease, particularly in males. However, our study did not reflect the unusually high frequency of Raynaud’s phenomenon observed in previous studies. Only 10% of our patients presented with Raynaud’s phenomenon, showing no preference for any AFD variant or the male sex, which is typically more susceptible to severe microcirculatory involvement. This discrepancy could be influenced by ERT, which was administered to 50% of our total sample and 80% of the male patients [45]. The differences between our findings and those of Deshayes et al. might be attributed to a higher male percentage in their control group or the improved microcirculation following treatment.

Our study, to the best of our knowledge, is the first to investigate capillaroscopic anomalies in different mutational variant subgroups of Anderson–Fabry disease, confirming the utility of capillaroscopy in early diagnosis and clinical management. Notably, differences in capillaroscopic alterations between sexes were observed, specifically in neovascularization and average capillary length, paving the way for further wider studies focusing on sex differences and macro- and microvascular complications.

The nailfold capillaroscopy method is well suited for global clinical application, thanks to the standardization of protocols and the reliability of the technique. Studies have shown that, with appropriate training and the use of standardized guidelines, capillaroscopic assessments can be consistently reproduced across different clinical settings and by doctors with varying levels of experience. The introduction of automated systems further enhances the objectivity and consistency of evaluations, reducing human error and making the process faster and more reliable. Additionally, the use of standardized terminology and evaluation criteria, as recommended by the EULAR Study Group, ensures that the same reference values can be applied worldwide, facilitating consistent and comparable results. This consensus in methodology underscores the global applicability of NC, enabling its use as a reliable tool in different clinical environments [48,49,50,51,52].

This study introduces capillaroscopy as a new tool for early diagnosis and continuous monitoring of Anderson–Fabry disease, highlighting the correlation between increased carotid IMT and Lyso-Gb3 accumulation. It recommends particular attention to certain capillaroscopic findings in the context of specific GLA gene mutations (classic, late-onset, and VUS).

An exploratory analysis according to patients’ treatment status revealed a statistically significant difference between the two groups only in the presence of the “sub-papillary plexus”, without emerging differences in other parameters or vascular complications. While the data suggest that ERT may affect specific microcirculatory parameters like the “sub-papillary plexus”, it does not seem to significantly influence the broader range of capillary metrics or clinical complications. The current findings are limited by the small sample size, the heterogeneous duration of ERT among patients, which may have influenced the results and limits the generalizability of our findings, and the distribution of patients across therapies—specifically, with only 10 patients on ERT and 4 on migalastat. This fragmentation makes it difficult to draw definitive conclusions about the broader efficacy of these treatments on microcirculatory parameters and clinical outcomes. The observed trends, particularly regarding the presence of the sub-papillary plexus, suggest potential areas of impact, but further research with larger, more homogeneous patient groups is necessary to fully understand the clinical implications of these therapies. Clinically, this underscores the need for personalized treatment approaches and highlights the importance of closely monitoring microcirculatory changes in patients undergoing these therapies to optimize outcomes.

Overall, this study has limitations including a modest sample size (due to the rarity of the disease), potential confounding factors (connective tissue diseases, diabetes mellitus), and technical difficulties (reproducibility of exams over time, patient availability). Future studies should aim to confirm our findings with larger cohorts and explore the long-term benefits of early diagnosis and treatment.

## 5. Conclusions

In conclusion, our study confirms the utility of capillaroscopy in diagnosing and managing Anderson–Fabry disease. By highlighting specific capillaroscopic findings in the context of different GLA gene variant subgroups (classic, late-onset, and VUS), our research proposes capillaroscopy as a valuable tool for early diagnosis and continuous monitoring. Further studies are necessary to validate these findings and explore new therapeutic strategies for AFD patients.

## Figures and Tables

**Table 1:** Characteristics of study population in the three subgroups according to the GLA variant.

	CLASSIC (*n* = 7)	LATE ONSET (*n* = 10)	VUS (*n* = 8)	*p*-Value
Age, yo	42.4 ± 14.7	42.2 ± 16	42.7 ± 15.8	0.998
Males, *n* (%)	5 (71.5)	2 (20)	4 (50)	0.139
alpha-gal-A (nmol/mL/h)	3.37 ± 4.35	9.39±4.98	9.93 ± 2.21	0.070
Lyso-GB3 (nmol/L)	21.6 (14–29)	1.4 (0.8–1.75)	1.3 (1–1.65)	0.002
Average Length				
Normal	1	7	4	0.043
Increased	0	3	1	0.223
Reduced	7	0	2	<0.001
Flow				
Visible	0	3	2	0.229
Sludge	7	1	3	0.004
Not visible	1	6	2	0.090
Loop Tortuosity				
<20%	5	0	1	0.008
20–50	2	9	5	0.009
>50%	1	1	0	0.683
Ectasia				
<10%	3	6	3	0.605
10–30	0	1	1	0.570
30–50	1	3	0	0.239
>50%	4	0	3	0.037
Sub-papillary plexus				
Visible	4	4	1	0.336
Not visible	1	6	6	0.012
Dilated	3	0	0	0.027
Giant Capillaries				
Diffuse	2	0	0	0.099
Neoangiogenesis				
Absent	6	4	7	0.029
Limited	0	1	0	0.458
Moderate	2	5	0	0.076
Microhemorrhages				
Absent	4	7	5	0.605
Rare	3	2	2	0.713
Diffuse	1	1	0	0.643
Edema				
Absent	4	4	4	0.777
Moderate	3	5	1	0.318
Marked	1	1	2	0.559
Acrosyndrome				
Absent	4	6	3	0.214
Complex	0	1	2	0.506
Raynaud	1	0	2	0.423
Pseudovascular Areas				
Absent	3	2	1	0.600
Limited	5	8	4	0.562
Diffuse	0	0	2	0.061
Avascular Areas				
Focal Absence	1	2	2	0.836
Dishomogeneity				
Absent	1	0	2	0.165
Moderate	7	7	4	0.395
Marked	0	3	1	0.223
Angiotectonic Disorder				
Absent	1	0	2	0.203
Moderate	6	8	4	0.571
Marked	1	2	1	0.902

**Table 2:** Intima-media thickness in left and right carotid in the three subgroups.

	CLASSIC (*n* = 7)	LATE ONSET (*n* = 10)	VUS (*n* = 8)	*p* Value
IMT Left CCA	0.812	0.634	0.715	0.236
IMT Right CCA	0.892	0.606	0.692	0.012

## Data Availability

The data underlying this article will be shared on reasonable request to the corresponding author.
